# Decoding pediatric spinal tumors: a single-center retrospective case series on etiology, presentation, therapeutic strategies, and outcomes

**DOI:** 10.1007/s10143-024-02770-w

**Published:** 2024-09-06

**Authors:** Pavlina Lenga, Daniel Kühlwein, Martin Grutza, Mohammed Issa, Felix Hinz, Felix Sahm, Florian Selt, Till Milde, Patrick Günther, Andreas W. Unterberg, Sandro M. Krieg, Ahmed El Damaty

**Affiliations:** 1 Department of Neurosurgery, Heidelberg University Hospital, Heidelberg, Germany; 2 Department of Neuropathology, Institute of Pathology, Heidelberg University Hospital, Heidelberg, Germany; 3 Hopp Children’s Cancer Center Heidelberg (KiTZ), Heidelberg, Germany; 4 Clinical Cooperation Unit Pediatric Oncology, German Cancer Research Center (DKFZ) and German Consortium for Translational Cancer Research (DKTK), Heidelberg, Germany; 5 National Center for Tumor Diseases (NCT), Heidelberg, Germany; 6 Department of Pediatric Hematology and Oncology, Heidelberg University Hospital, Heidelberg, Germany; 7 Department of General, Visceral and Transplantation Surgery, Division of Pediatric Surgery, Heidelberg University Hospital, Heidelberg, Germany; 8 Department of Neurosurgery, University of Heidelberg, Im Neuenheimer Feld 400, 69120 Heidelberg, Germany; 9 Present Address: Department of Pediatrics and Adolescent Medicine, University Hospital Jena, Friedrich Schiller University, Jena, Germany

**Keywords:** Pediatric spinal tumor, Neurosurgical oncology, Surgical decompression

## Abstract

**Introduction:**

Spinal tumors (ST) often result in dire prognosis, carrying risks such as permanent paralysis, sensory loss, and sphincter dysfunction. Data on their incidence and etiology in pediatric populations are markedly scant. Our study investigates the etiology, clinical manifestation, treatment, and outcomes of pediatric ST.

**Methods:**

We conducted a retrospective review of our institutional pediatric oncology and neurosurgery database, examining 14 patients under 18 years admitted with ST due to oncological diseases since 2005. We analyzed the clinical presentations, evaluations, molecular diagnostics and treatments for these patients.

**Results:**

The study spanned 15 years and included 14 pediatric patients, each diagnosed with distinct spinal tumor entity. The mean patient age was approximately 19.6 ± 10.1 months. Severe axial pain along the vertebral column was observed in 13 patients, while acute neurological deterioration manifested in 7 patients. As a first-line intervention, 13 patients underwent decompressive surgery through laminectomy and tumor resection, and only one patient received chemotherapy solely. Before surgery, seven patients were unable to walk; post-surgery, six of them regained their ability to ambulate. The diagnosis encompassed a range of neoplasms: two instances of Ewing sarcoma, 3 instances of teratoma, one case presenting an atypical teratoid Rhabdoid tumor, two instances each of low-grade astrocytoma and neuroblastoma, and single instances of ependymoma, meningioma, rhabdomyosarcoma, and embryonal tumors with multilayered rosettes (ETMRs). Three patients succumbed two years after initiating therapy.

**Conclusion:**

Despite their rarity, intraspinal tumors in pediatric patients pose substantial therapeutic challenges. The intertwined complexities of the disease entity and the patient’s neurological status demand swift initiation of an individualized therapeutic strategy. This crucial step helps optimize outcomes for this patient cohort, who frequently grapple with debilitating health conditions. Inclusion of these patients within a registry is mandatory to optimize treatment outcomes due to their rarity in pediatric population.

## Introduction

Spinal cord compression (SCC) or cauda equina syndrome is a formidable and fear-inducing condition arising in the realm of pediatric spinal malignancies, demanding urgent attention and critical management [5, 23]. Depending on the tumor’s anatomical position, SCC is taxonomically bifurcated into three distinct subcategories: extradural, intradural-extramedullary, and intramedullary. Unlike the adult population, the therapeutic strategy for malignant pediatric tumors resulting in spinal cord compression is meticulously customized according to the specific tumor type. Acute spinal cord or cauda equina compression, though relatively rare in pediatric patients with prevalence estimates ranging from 3 to 5% [16, 17], mandates immediate detection and intervention, typically initiated with magnetic resonance imaging (MRI) and an expeditious commencement of therapy [16, 17]. The rapid onset of neurological deterioration underscores an urgent therapeutic requirement, optimally within 24 h, to prevent, mitigate, or potentially reverse morbidity rates.

Despite the urgency of the situation, there exists a persistent academic debate regarding the initial line of therapeutic intervention for these debilitating cases - surgical intervention, radiation, or chemotherapy. Past studies have put forth the idea that surgical resection may serve as a cornerstone treatment in the context of intramedullary tumors [5, 11]. Conversely, the evidentiary basis for extramedullary and extradural tumors remains contentious and inconclusive, thereby warranting further investigation [8, 16, 28]. Given the varying strands of evidence and ensuing controversies, we assert an exigent need for further elucidation of optimal therapeutic approaches for pediatric populations afflicted with spinal tumors (ST), with the ultimate goal to enhance both the prognosis and quality of life for these young patients.

In this light, our study aims to examine the clinical progression of ST in the pediatric population, particularly those presenting with acute neurological symptoms, and are undergoing microsurgical decompression. We aim to scrutinize the etiological factors, assess associated morbidity and mortality rates, and evaluate long-term clinical outcomes following intervention as well as to provide a comprehensive overview on this subject.

## Methods

### Study design and inclusion criteria

Clinical and imaging data were retrospectively collected over a 16-year period (September 2005–December 2021) from our institutional database in a single center non-interventional study. This study was approved by the local ethics committee of our institution (approval number S307/2023) and conducted in accordance with the Declaration of Helsinki. Informed consent from patients’ parents or guardians were collected. This case series has been reported in line with the PROCESS Guideline 2020 [1]. Patients aged ≤18 years with histologically confirmed ST across the spinal cord or cauda equina as showed in MRI were consecutively enrolled. The exclusion criteria were as follows: age > 18 years; concurrent intracranial or cervical pathology, and unavailable data.

### Patient characteristics

Patient demographics, comorbidities, duration of surgery, number of treated spinal levels, perioperative and postoperative complications, hospital length of stay (LOS), intensive care unit (ICU) stay, readmission, reoperation, and mortality were retrieved from patients’ electronic medical records.

The degree of motor deficit was evaluated by prospectively applying the Spinal Injury Association Impairment Scale adapted to patients’ age [20]. It was graded as follows: grade 1, mild hyposthenia with walking disability for legs, or difficulty in raising hands above head for arms; grade 2, moderate hyposthenia with inability to walk and make movements against gravity or raise the hands above the head; grade 3, severe hyposthenia with paraplegia, no elicitable tendon reflexes or muscular movements. Patients presenting acute neurological decline underwent posterior decompression via laminectomy in the first 24 h. Solely one patient underwent conservative management. Decision making was guided by presenting neurological status, concomitant underlying pathologies, extent of the pathology, prognosis of the disease, and the discretion of an experienced treatment team of neurosurgeons, neuroradiologists, pediatric neurologists and pediatric neurooncologists. In case of sacral teratomas with extra-spinal pelvic extension, the resection was performed in cooperation with pediatric surgeons. Additionally, neuropathological and molecular diagnostics were thoroughly examined. Postoperatively, our patients were placed on a carefully tailored care plan that included immediate mobilization, as per the latest evidence supporting early post-surgical activity to enhance recovery outcomes. Mobilization began under the supervision of our pediatric physiotherapy team within 24 h after surgery, contingent on the patient’s stability and specific surgical details.

### Surgical procedures

The surgeries were conducted by a dedicated team of experienced neurosurgeons: S.K., A.E., and A.U., each of whom has over 20 years of specialized experience in pediatric neurosurgery. This team was specifically chosen for each procedure based on their expertise with the particular type of spinal pathology being addressed. Surgical decompression was primarily achieved through laminectomy, with tumor excision being performed extra- or intradurally based on the specific pathology encountered. Each surgical procedure was tailored to the individual patient’s needs, taking into account the location and severity of the tumor. The duration of surgeries varied based on the complexity of the case, with a range of 2 to 6 h. Detailed timing of each phase of the surgery was meticulously recorded to provide a comprehensive timeline of the intervention. Postoperative care commenced immediately in the pediatric intensive care unit (ICU), with patients being closely monitored for neurological status and recovery progress. The length of stay in each care phase was determined by the patient’s individual response to surgery and recovery needs.

#### Assessment of sensory deficits and pain levels

In this study, sensory deficits in pediatric patients were quantitatively assessed using the Pediatric Modified Rankin Scale (PMRS), a standardized tool designed to evaluate neurological function and recovery. This scale provides a systematic approach for measuring sensory impairment and improvements in children, ensuring consistent and reproducible results across different time points. For the evaluation of pain levels, we employed the Wong-Baker FACES Pain Rating Scale. This scale is specifically tailored for young children, featuring a series of facial expressions that correspond to different pain intensities, enabling patients to visually communicate their pain levels. The use of these validated tools ensures that our measurements of sensory and pain outcomes are both objective and reliable, suitable for the pediatric population involved in our study.

The detection of bladder and sphincter dysfunction in very young children was conducted through an integrated approach combining clinical assessment and caregiver reports. Pediatric patients were evaluated by pediatric neurologists and specialized nursing staff for clinical signs of urinary incontinence, urgency, frequency, constipation, and fecal incontinence. These signs were monitored both during hospitalization and through follow-up consultations.To augment clinical observations, parents and caregivers were provided with a structured reporting form designed to log any abnormal urinary or bowel patterns observed at home. This tool was crucial for capturing symptoms that might manifest outside of clinical settings, particularly in infants and toddlers who cannot verbally express discomfort.

#### Follow-up protocol

Post-operative follow-up examinations were systematically conducted in accordance with our institution’s established protocols for pediatric spinal tumors. Initial post-operative evaluations occurred before discharge, performed by a team of pediatric neuro-oncologists and neurosurgeons. These evaluations included comprehensive neurological assessments and imaging studies to assess immediate surgical outcomes and neurological function. Subsequent follow-ups were scheduled at three months post-surgery, involving detailed clinical and imaging assessments to monitor patient recovery and tumor response. Beyond the initial three-month follow-up, subsequent evaluations were guided by institutional oncological protocols, which are derived from current clinical guidelines. These follow-ups were designed to track long-term outcomes, detect any recurrence, and manage ongoing patient care. This standardized approach ensured consistency across all patient assessments and adherence to the highest standards of pediatric oncological care.

### Statistical analysis

Categorical variables were presented as numbers and percentages. Continuous variables are presented as mean ± standard deviation, and the Shapiro–Wilk test was used to verify whether their distribution was normal. Surgical characteristics and complication rates were compared groupwise using independent t-tests for continuous variables and chi-squared tests for categorical variables.

## Results

### Epidemiological data and baseline characteristics

**Fig. 1:**
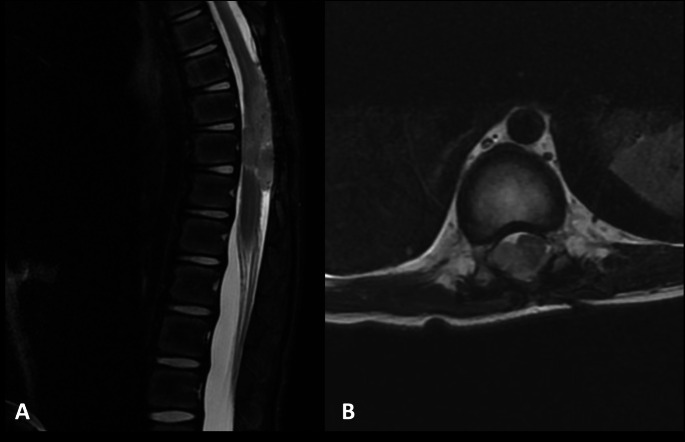
Sagittal (**A**) and Axial (**B**) MRI T2 Sequences Displaying Anaplastic Ependymoma in the Thoracic Spine of a 6-months-Old (ID_10). The pronounced compression of the cauda equina is evident. Remarkably, the child exhibited severe spinal pain without accompanying neurological deficits. Surgical intervention involved a laminectomy for tumor resection

**Fig. 2:**
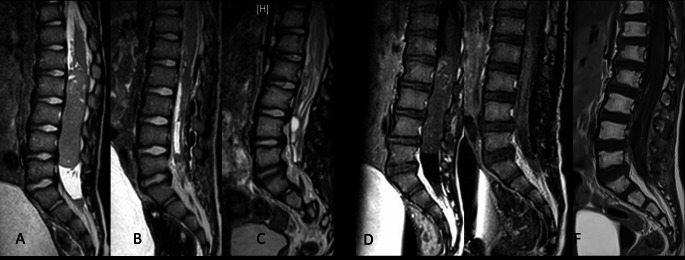
Preoperative (**A**, **D**), postoperative (**B**, **E**), and one-year follow-up (**C**, **F**) MRI images, both sagittal T2 and with contrast, show a spinal ETMR located in the lumbar spine of a 28-months-old child (ID_04). This child experienced severe pain, controllable only with morphine, and acute paraplegia. An urgent laminotomy from L1 to L5 and tumor resection were performed, resulting in total removal of the tumor. This was followed by multimodal adjuvant therapy

Spanning a duration of 15 years, this study involved 14 pediatric patients, each diagnosed with varied spinal tumor entity. The average age of the participants was approximately 19.6 ± 10.1 months. Notably, acute neurological deterioration was observed in seven patients. More specifically, two patients exhibited a grade 2 motor deficit, while five patients presented with a grade 3 motor deficit. Dysfunction of the bladder and sphincter were seen in three patients, respectively. Likewise, respiratory distress was reported in three cases. An analysis of the tumor locations revealed a predominance in the thoracic and lumbar spine, with respective prevalence rates of 50.0% and 28.6%. Soft tissue sarcoma emerged as the most common tumor entity, represented in 28.6% (4 out of 14) of the cases. Moreover, extradural compression was notably present in half of the cases (7 out of 14, 50.0%). A detailed depiction of patient characteristics is provided in Table 1. Representative images of two cases are presented by Figs. 1 and 2.

**Table 1:** Baseline characteristics

Characteristic	Value	
Number of patients	14	
Age, months (mean, SD)	19.6 (10.1)	
Sex (n, %)		
Male	7 (50.0)	
Female	7 (50.0)	
Body mass index, kg/m^2^ (mean, SD)	16.3 (3.1)	
Symptoms (n, %)		
Motor Deficit *	7 (50.0)	
Grade 1	0 (0.0)	
Grade 2	2 (14.3)	
Grade 3	5 (35.7)	
Pain	14 (100.0)	
Bladder Dysfunction	3 (21.4)	
Sphincter Dysfunction	3 (21.4)	
Respiratory distress	2 (14.3)	
Level of Spinal Cord Compression (n, %)		
Cervical	2 (14.3)	
Thoracic	7 (50.0)	
Lumbar	4 (28.6)	
Lumbosacral	1 (7.1)	
Tumor entity (n, %)		
Neural tumors	2 (14.3)	
Soft-tissue sarcoma	4 (28.6)	
Ependymal cell tumors	1 (7.1)	
Germ cell tumor	1 (7.1)	
Glial tumor	2 (14.3)	
Meningeoma	1 (7.1)	
Location of tumor (n, %)		
Intramedullary	2 (14.3)	
Intradural extramedullary	5 (35.7)	
Extradural	7 (50.0)	

*According to Spinal Injury Association Impairment Scale

### Surgical characteristics, clinical course and complications

This study comprised 13 patients who initially underwent microsurgical decompression with tumor debulking, while one patient was subjected to conservative management. As detailed in Table 2, the mean duration of surgery stood at approximately 219.8 ± 110.3 min, accompanied by an average blood loss of approximately 210.5 ± 102.3 ml. The mean number of levels decompressed was approximately 1.9 ± 1.2. Unreassuringly, one patient succumbed to the progression of sarcoma during hospitalization.

**Table 2:** Peri- and postoperative surgical characteristics and clinical course of the 13 pediatric patients who underwent decompression surgery with respect to the anatomic compartments

Characteristic	All (*n* = 13)	Intradural (*n* = 7)	Extradural (*n* = 6)	P*
Surgical duration, minutes	219.2 (110.3)	293.0 (90.1)	232.9 (54.2)	0.247
Number of levels decompressed	1.9 (1.2)	1.5 (0.5)	2.2 (0.3)	0.369
Hospital stay, days	17.0 (8.5)	13.4 (7.5)	20.0 (7.1)	0.082
ICU stay, days	0.8 (1.6)	0.4 (0.5)	1.2 (2.2)	0.945
Mortality				
In-hospital (n, %)	1 (7.7)	0 (0.0)	1 (16.7)	0.461
90-day (n, %)	1 (7.7)	0 (0.0)	1 (16.7)	0.462
30-day readmission (n, %)	2 (15.4)	1 (14.2)	1 (16.7)	0.261
Motor deficits after surgery (n, %)				0.556
Grade 1	2 (15.4)	1 (14.2)	1 (16.7)	
Grade 2	4 (30.8)	2 (28.5)	2 (33.3)	
Grade 3	1 (7.7)	0 (0.0)	1 (16.7)	
Chemotherapy after surgery (n, %)	9 (69.2)	4 (57.1)	5 (83.3)	0.725
Radiation after surgery (n, %)	7 (53.8)	4 (57.1)	3 (50.0)	0.797

The values are indicated as mean (SD) unless otherwise indicated

ICU, intensive care unit; MS, motor score of the American Spinal Injury Association grading system

*Comparison between intra-and extradural pathologies

Postoperative outcomes indicated a substantial improvement in the neurological status of the patients, with only one instance of a grade 3 motor deficit). In the second stage of our analysis, we compared patients post-surgery based on the anatomical location of their tumors. Intriguingly, we observed no significant differences between the groups in terms of surgical characteristics, outcomes, or complication rates (Tables 2 and 3).

**Table 3:** Occurrence of adverse events in patients who underwent decompression surgery with respect to the anatomic compartments

Event	All (*n* = 13)	Intradural (*n* = 7)	Extradural (*n* = 6)	P*
Deep wound infection	1 (7.7)	0 (0.0)	1 (16.7)	0.789
Epidural hematoma	1 (7.7)	0 (0.0)	1 (16.7)	0.565
Cerebral fluid leakage	2 (15.4)	2 (28.5)	0 (0.0)	0.261
Revision surgery	4 (30.8)	2 (28.5)	2 (33.3)	0.899

*Comparison between intra-and extradural pathologies

Complications were reported in four patients, all of whom subsequently underwent revision surgery, as outlined in Table 3. Of note, before surgery, seven patients were unable to walk; while post-surgery, six of them regained their ability to ambulate. Table 4 provides a detailed overview of the neurological status of patients before and after surgery. One patient, diagnosed with neuroblastoma devoid of any neurological deficits or significant compression of the epidural space of the spinal canal, was subjected exclusively to conservative management with chemotherapy.

**Table 4:** Neurological function Assessment: Pre-surgery vs. Post-surgery comparison

ID	Age on admission in months	date of surgery	Pathology	Spinal Level	Neurological Status before surgery	Neurological status at discharge
ID_01	6	2013	Low grade astrocytoma WHO grade 1	C3-C7	Motor and sensory paraparesis (KG 2/5)	Paraparesis (KG 4/5); able to walk
ID_02	18	2015	pilocytic astrocytoma WHO grade 1	Th10-Th12	Paraparesis affecting the lower extremities (KG 4/5), accompanied by sensory deficits in temperature perception	Complete recovery from the motor loss; persisting sensory deficits
ID_03	16	2018	atypical meningeoma WHO grade 2	C3-C5	Neurogenic voiding and sphincter disorder accompanied by concurrent leg pain	Improvement; able to walk
ID_04	28	2018	embryonal tumor with multiplane rosettes	L1-L5	Right-sided sciatica radiating down the right thigh and calf, acute paraplegia (KG 0/5)	Improvement of pain levels, able to walk
ID_05	19	2018	metastasis of an Ewing sarcoma	Th 7-Th10	Motor and sensory paraplegia in the lower extremities (KG 0/5)	Paraparesis (KG 3/5) in the lower extremities; able to walk with assistance
ID_06	1	2019	unmature teratoma	L3-L4	Pain, Sphincter dysfunction	Improvement of pain levels
ID_07	1	2019	mature cystic teratoma (G0)	S1-S2	Motor and sensory paraplegia in the lower extremities (KG 0/5)	Paraparesis in lower extremities (KG 3+/5): able to walk with assistance
ID_08	15	2021	alveolar rhabdomyosarcoma	Th5-Th6	Paraparesis in the lower extremities (KG 3/5)	Complete recovery; able to walk
ID_09	13	2021	Ewing sarcoma	Th6-Th8	Voiding and sphincter dysfunction accompanied by sensory disorders in both legs	Slight improvement of sensory deficits
ID_10	6	2021	anaplastic ependymoma WHO grade 3	Th9-Th10	Respiratory distress, no deficits	Improvements
ID_11	25.5	2021	low differentiated neuroblastoma	Th7-Th10	Voiding disorder; gait disturbance	No change
ID_12	2	2022	malignant atypical teratoid/rhabdoid tumor, subclass MYC, WHO grade 4	Th7-L1	Motor and sensory paraparesis in the lower extremities (KG 2/5)	Paraparesis (KG 4+/5); able to walk
ID_13	50	2023	vital and mature teratoma	S2-S5	Motor and sensory paraplegia in the lower extremities, sphincter dysfunction (KG 0/5)	Improvement of paraparesis in lower extremities (KG 2/5): not able to walk

KG: Kendall grading system for evaluation of muscle strength

The diagnosis distribution was as follows: three patients with Ewing sarcoma, 3 with teratoma, 1 with Atypical Teratoid Rhabdoid Tumor (ATRT), two each with low-grade astrocytoma and neuroblastoma, and the remainder diagnosed with ependymoma, meningioma, and rhabdomyosarcoma, respectively. Table 5 provides a concise overview of the neuropathological diagnoses. DNA-methylation classification was done all (6/6) CNS tumor cases. The mean follow-up period was approximately 19.8 ± 5.6 months. During this interval (two years after therapy), three patients diagnosed with Ewing sarcoma unfortunately passed away due to disease progression.

**Table 5:** Histological and molecular findings across 14 pediatric patients with spinal tumors n.d.: not determined

ID	Age on admission in months	date of surgery	histological diagnosis	molecular diagnosis (classifier score; classifier version)
ID_01	6	2013	Low grade astrocytoma WHO grade 1	methylation class Diffuse astrocytoma, MYB or MYBL1-altered, subtype B (0.99; v12.8)
ID_02	18	2015	pilocytic astrocytoma WHO grade 1	no match to an established reference class (< 0.3; v12.8)
ID_03	16	2018	atypical meningeoma WHO grade 2	methylation class Meningioma, subtype benign, subclass 1 (0.8; v12.8)
ID_04	28	2018	no known CNS tumor or sarcoma, most probable embryonal tumor with multiplane rosettes	methylation class Embryonal tumor with multilayered rosettes, non-C19MC-altered (novel) (0.97; v12.8)
ID_05	19	2018	metastasis of an Ewing sarcoma	n.d.
ID_06	1	2019	unmature teratoma G1 after Gonzales-Crussi (includes lung, dermal, mesenchymal, smooth muscles, neuronal, vascular, adipose and cartilage tissue)	n.d.
ID_07	1	2019	mature cystic teratoma (G0)	n.d.
ID_08	15	2021	alveolar rhabdomyosarcoma	n.d.
ID_09	13	2021	Ewing sarcoma	n.d.
ID_10	6	2021	anaplastic ependymoma WHO grade 3	no match to an established reference class, highest score for methylation class family Posterior fossa ependymoma group A (0.79; v12.8)
ID_11	25.5	2021	low differentiated neuroblastoma	n.d.
ID_12	2	2022	malignant atypical teratoid/rhabdoid tumor, subclass MYC, WHO grade 4	methylation class Atypical teratoid/rhabdoid tumor, MYC-subtype (0.99; v12.8)
ID_13	50	2023	vital and mature teratoma	n.d.
ID_14	74	conservative	neuroblastoma	n.d.

### Illustrative case 1

In our study, we closely examined the case of an 19-month-old patient (ID_05) diagnosed with Ewing’s sarcoma presenting with high-grade paraparesis and significant neurological deficits upon admission. Magnetic resonance imaging (MRI) identified a dumbbell-shaped extradural tumor along the thoracic spine. The tumor exerted substantial compression on the dural sac and extended to extraspinal areas through the spinal foramina. This severe presentation necessitated rapid and decisive intervention. Recognizing the critical nature of the patient’s condition, emergency surgery was executed within 24 h of admission. A laminectomy was performed at the thoracic levels T7 to T10 to access and decompress the spinal canal. Following the laminectomy, careful and meticulous tumor resection was conducted. The procedure focused on subtotal removal of the extradural mass, which was crucial for alleviating the compression on the dural sac and stabilizing the neurological function. The tumor’s extension through the spinal foramina was addressed by carefully dissecting the tumor from the surrounding tissues, ensuring minimal disturbance to the spinal nerves. Postoperatively, the patient demonstrated a remarkable improvement in mobility, with motor grades improving to KG 3/5. Despite the initial postoperative improvements, consistent with prevailing practices in the treatment of high-grade spinal tumors, radiotherapy was administered to manage residual tumor mass and mitigate the risk of recurrence. However, the patient’s condition deteriorated, and they unfortunately passed away three months post-surgery.

### Illustrative case 2

A two-month-old patient (ID_12) was admitted with severe pain localized to the midline thoracic spine. The patient exhibited symptoms of motor and sensory paraparesis in the lower extremities, indicating significant neurological impairment. MRI was performed, revealing an extramedullary intradural lesion at the lumbar spine. This imaging finding was crucial for guiding the subsequent therapeutic approach. The lesion was located from Th7 to L1, encompassing the intradural extramedullary space. Given the severity of the symptoms and the location of the tumor, decompressive surgery was promptly conducted, followed by total resection of the tumor. The procedure began with a laminectomy from Th7 to L1, meticulously executed to expose the affected sections of the spine without causing additional trauma to the delicate spinal tissues. After achieving adequate decompression through the laminectomy, the next phase involved the meticulous total resection of the intradural tumor. The extramedullary intradural nature of the tumor allowed for a clear dissection plane, facilitating a complete resection with minimal risk of residual tumor left behind. This immediate intervention aimed to alleviate the pressure on the spinal cord, thus preserving remaining neurological function. Histopathological analysis confirmed the diagnosis of malignant Atypical Teratoid/Rhabdoid Tumor (ATRT), subclass MYC, WHO grade 4. This aggressive tumor necessitated a comprehensive treatment strategy. Post-surgery, the patient began a regimen of chemotherapy aimed at targeting residual disease and preventing recurrence. Following the surgical and chemotherapeutic interventions, the patient showed a significant reduction in pain and some improvement in motor skills. Initially presenting with motor and sensory paraparesis (Kendall Grade (KG) 2/5), the patient’s condition improved to KG 4+/5; they were able to walk with assistance. After a year of treatment, the disease was observed to be stable, indicating a positive response to the interventions.

## Discussion

Acute Spinal Cord Compression (SCC) or cauda equina compression caused by tumorous process is an infrequent diagnosis in pediatric patients, presenting a prevalence of between 3 and 5%, and signifies a medical emergency that necessitates prompt initiation of therapy [16]. While the majority of the cases in this study involved significant spinal cord compression, there were exceptions, such as one case of neuroblastoma without significant epidural compression or neurological deficits. This highlights the diverse presentations and complexities of managing pediatric spinal tumors.

### Review of literature

#### Extradural spinal lesions

Ewing’s sarcoma constitute a relatively uncommon tumor category, accounting for approximately 3.5–9.8% of all Ewing’s sarcoma cases [12]. Until now, a limited number of such cases involving SCC (*n* = 65 across all age groups) have been reported, of which only ten were within the pediatric population [24, 26]. In terms of therapeutic interventions, surgical treatment is the primary approach for spinal neuroectodermal tumors. Its key benefits include preservation of functionality, pain alleviation, lesion removal, and more critically, the management of recurrence and the potential to prolong survival [4]. Prior studies have indicated that gross total resection may correlate with extended survival durations and improved functional outcomes in comparison to subtotal resection [29, 31]. A recent retrospective study conducted by Chen et al. on 40 patients with spinal ewing sarcoma, spanning all age groups, suggested that total en bloc resection resulted in significantly higher survival rates. Additionally, the study proposed that postoperative adjuvant radiotherapy could play a crucial role in enhancing survival outcomes [4]. It should be emphasized, however, that the subject pool of the study did not exclusively comprise the pediatric population. As a result, the best initial line of therapy remains an open question. In our study, focusing solely on pediatric spinal tumors, we encountered three patients with Ewing’s sarcoma who exhibited high-grade paraparesis of the lower extremities at the time of admission. MRI revealed a dumbbell-shaped extradural tumor along the thoracic spine, causing significant compression on the dural sac and extending to extraspinal areas through the spinal foramina. Emergency surgery was promptly executed within 24 h. Subtotal resection was performed in one case, while total tumor removal was achieved in the remaining two cases. All three patients exhibited notable improvements in mobility postoperatively. Consistent with previous studies, radiotherapy was subsequently administered. It is worth noting that the patient who underwent subtotal tumor removal unfortunately passed away three months post-surgery. We feel that gross total resection has the potential to optimize survival durations and maintain a high quality of life. Future comprehensive prospective studies are needed to refine treatment strategies and to provide a foundation for the establishment of guidelines for managing this significant yet rare disease.

Intraspinal teratomas within the pediatric population constitute a remarkably infrequent diagnosis, accounting for approximately 5–10% of cases [6]. Despite the gradually increasing incidence of intraspinal teratomas, the majority are found to be intra- or extramedullary in location [22]. According to the most comprehensive systematic review and meta-analysis of pediatric spinal teratomas to date, involving 170 cases, a mere 13 cases revealed spinal epidural positioning. The diagnostic process for these cases is challenging, primarily due to the variability in neurological deficits exhibited, ranging from nonspecific pain to motor weakness and gait disturbances [6, 22]. In the current study, three cases of teratoma were identified. As previously reported, patients initially presented with nonspecific pain along the spinal cord and skin stigmata, prompting MRI examinations that revealed tumor masses within the lumbosacral spinal cord. Microsurgical laminectomy and tumor removal, which is the standard of care [25], were performed successfully without perioperative or postoperative complications. Over a two-year follow-up period, no recurrence was noted. Based on our findings, we posit that when it comes to spinal teratomas, a meticulous clinical examination is indispensable for identifying potential diagnostic indicators. Moreover, immediate surgical removal is unequivocally essential to halt disease progression and to prevent neurological deterioration caused by significant epidural compression. Given the benign nature of these tumors, prompt surgical intervention emerges as a crucial management strategy for this disease. However, we underscore the importance of close patient monitoring. Regular follow-ups, including annual assessments with low-dose, full-length spine radiographs to monitor scoliosis progression, and yearly surveillance MRIs for a minimum of five years to detect potential disease recurrence, should be considered routine practice.

Neuroblastoma (NB), another distinct tumor type prevalent among the pediatric population, frequently manifests with spinal canal invasion, thereby often precipitating epidural compression. Such cases account for approximately 5-15% of all NB occurrences [2, 30]. The origins of neuroblastoma can typically be traced to the sympathoadrenal axis, with abdominal primaries being the most commonly reported, succeeded by thoracic and pelvic tumors. The spread of the tumor ensues through direct extension, lymphatic, and hematogenous dissemination. Within the scope of this discussion, we examine two instances of pediatric neuroblastoma marked by significant spinal canal invasion. In one instance, the patient manifested with an intensifying weakness in the lower extremities, coupled with bladder dysfunction. Consequently, a decompression of the spinal canal was executed via laminectomy and tumor resection. Post-surgical intervention, the patient underwent chemotherapy, in keeping with the current therapeutic recommendations, and showcased favorable neurological recovery. Conversely, the second patient exhibited no neurological symptoms, thus a conservative management approach involving exclusive chemotherapy was undertaken. Encouragingly, in both instances, tumor recurrence was absent during a follow-up period of two years. Our observations lend weight to the ongoing discourse regarding the initial choice between surgery or chemotherapy for the management of such cases. Historically, symptomatic Spinal Cord Injury (SCI) caused by tumor compression was managed primarily with decompressive neurosurgery and radiation therapy, prior to the realization of the efficacy of chemotherapy. Currently, radiation therapy is seldom employed for symptomatic malignant SCI. The current treatment guidelines for malignant SCI underscore the necessity of prompt referral to a specialist multidisciplinary team, encompassing pediatric oncology, neurosurgery, neurology, orthopedics, pathology, and radiology. This collaborative approach facilitates expedited clinical evaluation, along with diagnostic and staging investigations, and fosters efficient treatment planning and initiation. In symptomatic patients, immediate therapy initiation is deemed crucial. Treatment modality determination is influenced by a holistic consideration of the relative risks and benefits of each approach by the multidisciplinary team. While the primacy of initial chemotherapy is often emphasized, instances marked by acute neurological deterioration may necessitate surgical resection as a potentially curative measure and a means to alleviate the neurological deficit [30]. This once again underlines the necessity for an individualized, case-by-case approach in the management of such complex cases.

#### Intradural spinal lesions

Atypical Teratoid Rhabdoid Tumors (ATRT) are a rare phenomenon, comprising 1–2% of all pediatric brain tumors, with a frequency of 10–20% in patients under three years of age [3, 10]. Fewer than 50 primary spinal ATRT cases have been described in the literature, generally presenting with nonspecific pain and myelopathy due to significant spinal cord compression [19]. These symptoms necessitate a swift diagnosis and aggressive treatment regimen. In patients presenting with neurological deficits, surgical decompression and tumor resection are the preferred therapeutic modalities for histological diagnosis and prevention of further neurological deterioration [19]. Survival rates are somewhat uncertain, varying from a few weeks to up to five years post-diagnosis [14, 19]. Regardless of the chosen adjuvant therapy, most patients remain at a high risk for recurrence or progression within six months of treatment [3]. Li et al. (2019) presented the most extensive study on ATRT in the pediatric population to date, reporting on four patients who experienced pain and neurological deficits attributable to the level of spinal cord nerve root compression [19]. These patients underwent decompressive surgery, followed by chemotherapy and radiation. In our study, we report a two-year-old patient with ATRT, who experienced severe pain located at the spinal cord. MRI imaging revealed an extramedullary intradural lesion in the lumbar spine. Following decompressive surgery and tumor total resection, there was a significant reduction in the patient’s pain levels. Histological analysis confirmed the ATRT diagnosis, initiating a course of chemotherapy. After a year, we observed stable disease in the patient. Given these findings and extant literature, it seems that surgical decompression might be the first line therapy to preserve neurological function, whereas adjuvant radiation and chemotherapy are mandatory.

Embryonal tumors with multilayered rosettes (ETMRs) represent another class of aggressive and rare entities, primarily diagnosed in the pediatric population. These tumors are known for their association with poor prognosis [13]. ETMRs can develop in both supratentorial and infratentorial regions of the brain, with cerebral hemispheres being the most common sites of occurrence. These tumors often involve the frontal and parietotemporal regions. However, scant literature exists regarding their presence in the spinal cord, and effective management strategies remain elusive. In our study, we report on a 28-month-old patient who presented with acute paraplegia of the lower extremities. MRI findings indicated an intradural extramedullary lesion causing substantial compression of the spinal cord. Given the emergent nature of the case, we opted for immediate decompressive surgery with tumor debulking. Postoperatively, the patient was closely monitored in the ICU for 24 h, where we noted a significant improvement in the motor deficits. A course of chemotherapy was subsequently initiated, and the patient survived for 17.6 months post-surgery, as suggested by previous prospective studies [13, 15]. Aligned with the findings of Khan et al. and Juhnke et al. we noticed that surgical decompression coupled with chemotherapy is crucial for prolonging survival rates and improving outcomes in patients with ETMRs. Further investigations and broader clinical trials are required to validate these findings and develop definitive treatment protocols.

One of the more challenging aspects within the realm of pediatric healthcare concerns the treatment of spinal astrocytomas. In this discourse, we delineate the circumstances of two particular cases involving spinal astrocytomas, both of which manifested with acute clinical exacerbation. Consequently, a combination of surgical decompression and tumor debulking emerged as the primary therapeutic strategy. This intervention resulted in notable enhancements in motor function. Nonetheless, it is imperative to underscore the current state of contention within the scientific literature with respect to the optimum therapeutic approach. Pervasive within the medical canon is the understanding that surgical intervention typically forms the cornerstone of therapeutic strategies for patients with intramedullary astrocytomas, particularly in instances involving neurological deficits or myelopathy. However, the approach’s efficacy remains less clear for cases primarily characterized by pain or incidental findings, as suggested by Hersh et al. [9]. The notion of gross total resection leading to increased free progression survival has been posited in earlier studies [7, 21]. Despite this, there may be an immediate requirement for the initiation of chemotherapy to circumvent the progression. In contrast, the use of radiation therapy has thus far been relatively limited in managing spinal astrocytomas in pediatric patients due to potential adverse effects, such as growth retardation, radiation necrosis, and vasculopathy. Parallel to the findings of the aforementioned studies, our management strategy also incorporated adjuvant chemotherapy due to residual tumor. This approach yielded encouraging results, with no discernible disease progression recorded throughout an extended follow-up period of over four years.

#### Challenges in decision making process

Despite significant advancements in oncological protocols, crucial gaps remain, especially in the management of spinal tumors in the pediatric population. These tumors are relatively rare in children, leading to the adaptation of adult protocols or the borrowing of strategies from similar tumors in other regions [3]. This extrapolation, while useful, may be suboptimal given the unique characteristics of pediatric patients and the tumors they present. Pediatric spinal tumors are heterogeneous, ranging from benign to highly malignant entities. The existing oncological protocols often group these diverse tumors together, complicating the development of individualized treatment strategies [10]. A significant limitation is the lack of randomized controlled trials (RCTs) due to the rarity of pediatric spinal tumors. As such, guidelines are primarily based on case reports, case series, or retrospective studies, each of which has inherent limitations [18, 19]. Aggressive treatment strategies, often required to manage these tumors, can have long-term effects on the quality of life of pediatric patients. Current protocols need refining to balance curative intent with the long-term well-being of these young patients [3]. In light of these identified gaps, this study, albeit limited, may serve as a basis for further research into this rare and challenging clinical entity. Our findings highlight the urgent need for additional high-quality research, including RCTs and longitudinal studies, to refine oncological protocols and improve management strategies and outcomes for these patients. Hence, the significance of registries, such as the Soft Tissue Sarcoma Registry, becomes evident. These registries are instrumental in the formulation of guidelines and the establishment of standardized treatment protocols. This systematic approach ensures that patients across all age demographics receive appropriate and effective treatment, thereby potentially enhancing survival rates [27].

### Limitations

The principal merit of this study lies in the systematic exploration of the clinical trajectory and outcomes pertaining to a range of rare tumor entities with spinal location confined to the pediatric demographic. This research was conducted on a relatively small patient cohort, which might initially appear as a limitation. However, given that extant data on these diseases predominantly emerge from case reports, we posit that our findings offer a comprehensive and pragmatic portrayal of these afflictions. It is important to acknowledge potential selection bias, which may have been introduced due to the retrospective design of the study. Additionally, the limited case numbers precluded the possibility of conducting a multivariate analysis for the loss of ambulation. This limitation underscores the imperative need for more extensive studies to shed light on the intricate mechanisms and roles of various therapeutic modalities. Such investigations are crucial for elucidating the optimal management strategies for these rare diseases, thereby enhancing the overall quality of pediatric healthcare.

## Conclusions

Spinal tumors in the pediatric population, while infrequent, constitute a considerable therapeutic conundrum. The disease entity including full diagnostic molecular workup, intertwined with the neurological status of the patient, necessitates the rapid initiation of a tailored therapeutic approach. This is key to optimizing the outcomes for this patient cohort, often grappling with debilitating health conditions. The pivotal elements for successful recovery in such patients encompass timely integrated neuropathological diagnosis, assertive treatment regimens, and rigorous monitoring. Future endeavors should focus on developing robust protocols and guidelines to aid in the efficient management of such rare diseases. This will expedite the management of epidural compression and curtail both the incidence and severity of long-term disabilities. Ultimately, the primary aim is to enhance the quality of life for these young patients, offering them the chance to reach their full potential in spite of the challenges posed by their diagnosis.

## Data Availability

No datasets were generated or analysed during the current study.
